# Structural Analysis and Activity Correlation of Amphiphilic Cyclic Antimicrobial Peptides Derived from the [W_4_R_4_] Scaffold

**DOI:** 10.3390/molecules28248049

**Published:** 2023-12-12

**Authors:** Shaima A. El-Mowafi, Anastasia G. Konshina, Eman H. M. Mohammed, Nikolay A. Krylov, Roman G. Efremov, Keykavous Parang

**Affiliations:** 1 Center for Targeted Drug Delivery, Department of Biomedical and Pharmaceutical Sciences, Chapman University School of Pharmacy, Harry and Diane Rinker Health Science Campus, Irvine, CA 92618, USA; sa.el-mowafi@nrc.sci.eg (S.A.E.-M.); emohammed@chapman.edu (E.H.M.M.); 2 Peptide Chemistry Department, National Research Centre, Dokki, Cairo 12622, Egypt; 3 M.M. Shemyakin & Yu.A. Ovchinnikov Institute of Bioorganic Chemistry, Russian Academy of Sciences, Miklukho-Maklaya Street, 16/10, 117997 Moscow, Russia; konnij@gmail.com (A.G.K.); krylovna@gmail.com (N.A.K.); 4 Department of Chemistry, Faculty of Science, Menoufia University, Shebin El-Koam 51132, Egypt; 5 Department of Applied Mathematics, National Research University Higher School of Economics, Myasnitskaya ul. 20, 101000 Moscow, Russia

**Keywords:** antimicrobial activity, molecular dynamics, membrane, peptide, structure-activity relationship

## Abstract

In our ongoing quest to design effective antimicrobial peptides (AMPs), this study aimed to elucidate the mechanisms governing cyclic amphiphilic AMPs and their interactions with membranes. The objective was to discern the nature of these interactions and understand how peptide sequence and structure influence antimicrobial activity. We introduced modifications into the established cyclic AMP peptide, [W_4_R_4_], incorporating an extra aromatic hydrophobic residue (W), a positively charged residue (R), or the unique 2,5-diketopiperazine (DKP). This study systematically explored the structure–activity relationships (SARs) of a series of cyclic peptides derived from the [W_4_R_4_] scaffold, including the first synthesis and evaluation of [W_4_R_4_(DKP)]. Structural, dynamic, hydrophobic, and membrane-binding properties of four cyclic peptides ([W_4_R_4_], [W_5_R_4_], [W_4_R_5_], [W_4_R_4_(DKP)]) were explored using molecular dynamics simulations within a DOPC/DOPG lipid bilayer that mimics the bacterial membrane. The results revealed distinct SARs linking antimicrobial activity to parameters such as conformational plasticity, immersion depth in the bilayer, and population of the membrane binding mode. Notably, [W_4_R_5_] exhibited an optimal “activity/binding to the bacterial membrane” pattern. This multidisciplinary approach efficiently decoded finely regulated SAR profiles, laying a foundation for the rational design of novel antimicrobial peptides.

## 1. Introduction

Amphipathic membrane-active peptides engage in selective interactions with a diverse range of membranes, utilizing various mechanisms to disrupt lipid bilayers [1]. These interactions depend on specific peptide attributes and the composition of the membrane. This class of compounds encompasses both cell-penetrating peptides (CPPs) and antimicrobial peptides (AMPs) [2].

CPPs, typically composed of 5–30 amino acids, have the capacity to transport a wide range of biomolecules into cells, including small drugs, small interfering RNA, proteins, peptide nucleic acid, imaging agents, and vaccines [2]. They hold promise as therapeutic agents and function as carriers for delivering therapeutic payloads across cell membranes. Because the most important factor determining the functioning of CPPs is related to their interaction with the cell membrane, the focus in studies of these peptides is on the delineation of the correlations between their structure/structure-based properties (e.g., amphiphilicity, charge, conformational flexibility) and the membrane activity. Such structure–activity relationships (SARs) are indispensable for the rational design of new CPPs with predefined properties [3,4]. These studies underscore the significance of optimizing SAR components for CPPs in enhancing their transfection efficacy. The same strategy also works in the case of membrane-active AMPs.

AMPs represent a class of peptides consisting of 5–50 amino acid residues that target bacterial membranes. AMPs typically have an excess of positively charged and hydrophobic residues, which are frequently separated, thus making the peptides highly amphiphilic. This property is important for the interaction between AMPs and membranes [5]. Shared characteristics with CPPs, such as amphipathicity or the presence of arginine-rich regions, suggest the potential for dual functionality for some of these molecules [6].

AMPs offer a promising alternative to conventional antibiotics in the treatment of drug-resistant pathogenic microbes [7,8]. They possess unique characteristics, such as distinct sequences, short length, cationic and amphiphilic characters, high affinity, and strong interaction with negatively charged bacterial cell membranes. Furthermore, they exhibit a broad spectrum of antimicrobial activity while preserving the integrity of host cells. The utilization of AMPs provides advantages such as their natural occurrence in combating infections and reduced likelihood of resistance development compared with traditional antibiotics [9].

The therapeutic applicability of AMPs is often constrained by their interaction with eukaryotic cell membranes, which can be detrimental to host cells. Researchers are thus focused on designing new AMPs with robust antimicrobial action and minimal cytotoxicity toward eukaryotic cells [10].

AMPs isolated from natural sources face various challenges, including low stability, limited salt tolerance, and high toxicity, impeding their widespread therapeutic application. Alterations in structural and physicochemical factors can impact the antimicrobial activity of these peptides. Therefore, studies of SAR profiles for both naturally occurring and artificially created peptides are very important and pave the way for the development of peptides with increased stability and a broad spectrum of action [11].

Cyclic AMPs have demonstrated enhanced membrane permeability compared with linear peptides. Specific amino acids play a pivotal role in influencing both the antibacterial action and cell cytotoxicity of AMPs. Peptides with increased net charge and hydrophobicity exhibit a heightened capacity to penetrate microbial membranes [12]. However, elevated hydrophobicity is often associated with increased mammalian cell toxicity and a loss of antimicrobial specificity [12].

**Figure 1:**
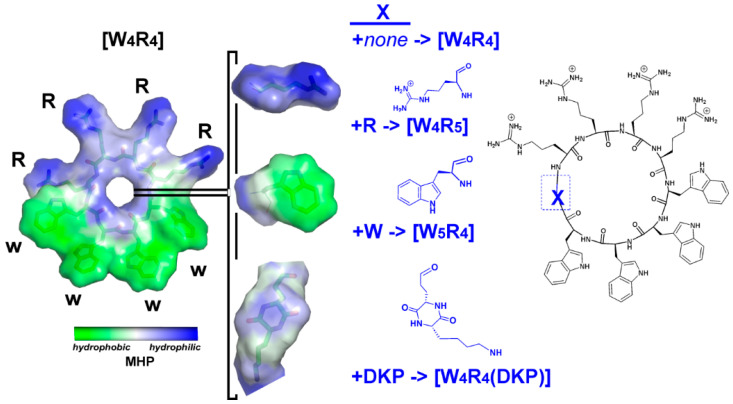
Chemical structures and hydrophobic/hydrophilic surface properties of [W_4_R_4_], [W_4_R_5_], [W_5_R_4_], and [W_4_R_4_(DKP)]. The insertion site of positively charged arginine, hydrophobic tryptophan, and weakly polar “DKP moiety” group is indicated with a blue rectangle in the chemical structure of the parent [W_4_R_4_], which is highly amphiphilic. The amphiphilic “DKP moiety” group includes the polar DKP ring and an apolar hydrocarbon chain. Surface properties of the molecules are color-coded according to the values of their molecular hydrophobicity potential (MHP, see the Section 4 (Methods) for details).

We have previously shown that amphiphilic cyclic peptide [W_4_R_4_] (Figure 1) containing both hydrophobic tryptophan (W) and positively charged arginine (R) residues acts as a promising lead AMP [13]. Recently, we reported synthesizing and evaluating cyclic peptides containing R and W residues with antimicrobial properties against multidrug-resistant and nonresistant Gram-positive and Gram-negative bacterial strains [13].

The accumulated extensive data on the antimicrobial activity of these cyclic peptides, along with insights into their potential undesirable properties such as hemolytic activity, presents an opportunity to elucidate the molecular mechanisms governing their actions and delineate the key SAR parameters. This understanding lays a robust groundwork for the further rational design of novel antimicrobial peptides that possess higher values of selectivity indices (SIs). Specifically, the challenge of optimizing SIs can be effectively tackled by adopting a comprehensive approach that integrates both experimental and computational methods.

As part of our ongoing efforts to design AMPs, we conducted a study investigating the mechanisms of several cyclic amphiphilic AMPs and their interactions with membranes. The objective was to elucidate the nature of the interaction and understand how the sequence and shape of the peptides influence antimicrobial activity. This was accomplished by introducing in the peptide [W_4_R_4_] either an additional aromatic hydrophobic residue (W), a positively charged residue (R), or a residue with “intermediate” properties-2,5-Diketopiperazine (DKP) (Figure 1). DKP was chosen due to its unique polar characteristics, making it an ideal residue for this modification. DKPs are the simplest naturally occurring cyclic peptides, commonly biosynthesized by a large variety of living organisms, including 90% of Gram-negative bacteria, Gram-positive bacteria, fungi, and higher marine organisms [14,15,16,17]. Since the earliest report on DKP in 1924 [18], they were found to span various biological activities. such as antitumor, antiviral, antifungal, and antibacterial activities, and to interfere with quorum sensing in bacteria [19,20]. The DKP scaffold, characterized by a six-membered ring, offers several advantages such as increased rigidity, enhanced resistance against enzymatic degradation, and the ability to form intermolecular H-bonds with target sites. These features make DKPs an attractive class of cyclic peptides with high potential as therapeutic agents. A comprehensive overview of their biological and structural profile has been recently published [21].

Thus, this study aims to investigate the structural and dynamic properties of cyclic peptides derived from [W_4_R_4_] with minimal modifications, incorporating additional aromatic hydrophobic (W) or polar positively charged (R) residues. This research seeks to understand the SARs of these peptides and their interactions with a model membrane. Specifically, this study introduces an “intermediate” substitution by incorporating DKP into the cyclic peptide [W_4_R_4_]. This approach combines peptide synthesis, in vitro antibacterial assays, and molecular modeling to inform the rational design of novel antimicrobial molecules with improved selectivity indices. This research also explores the binding of these peptides to a model membrane using molecular dynamics (MD) simulations and maps their hydrophobic properties to gain further insights into their interactions with the membrane.

## 2. Results

### 2.1. Design of This Study

In this study, we used peptide synthesis, in vitro biological testing, and molecular modeling to investigate the structural/dynamic and membrane-binding properties of a series of cyclic peptides obtained based on the [W_4_R_4_] template. To conduct a systematic SAR analysis, we made minimal changes to this peptide by incorporating a single W, R, or DKP residue, thus leading to peptides [W_5_R_4_], [W_4_R_5_], and [W_4_R_4_(DKP)], respectively (Figure 1). Importantly, the sequential arrangement of both residue groups within the cyclic structure was maintained. [W_4_R_4_] exhibited a SI of 43.8 that was calculated based on HC_50_/MIC against MRSA. Previous results by our laboratory [13] showed that these modifications in peptides [W_5_R_4_] and [W_4_R_5_] led to a decrease (SI = 12.5) and an increase (SI = 85.0) in their SI values, respectively.

Synthesis of [W_4_R_4_(DKP)] peptide (Figure 1) and the evaluation of its activity were performed in this study for the first time. The DKP moiety was formed with the intramolecular coupling reaction between the carboxylic acid side chain of glutamic acid and the amino group side chain of lysine. The resulting final cyclic peptide [W_4_R_4_(DKP)] was composed of two different rings, the cyclic [W_4_R_4_] scaffold encompassing the DKP ring. To elucidate the key components of SAR for these cyclic peptides for a high level of sequence and structure similarity, their binding to a model DOPC/DOPG membrane was studied with molecular dynamics (MD) simulations. Then, to gain further insights into their interactions with the membrane, we mapped the hydrophobic surface properties of the peptides. Based on the experimental and computational data, the SAR profiles of these peptides were derived and compared.

### 2.2. Chemistry

**Scheme 1:**
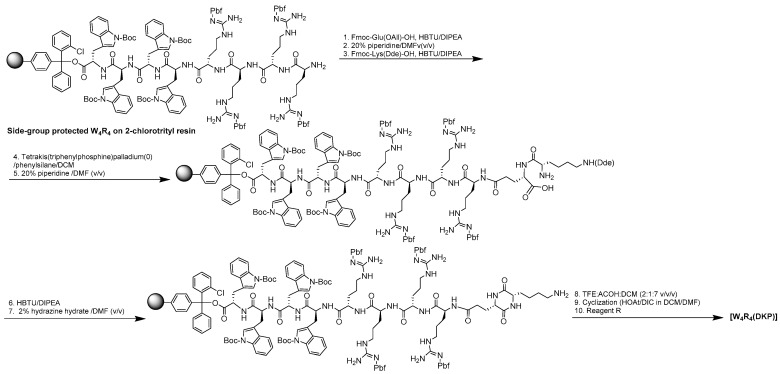
Synthesis of [W_4_R_4_(DKP)].

We previously reported the synthesis of [W_4_R_4_], [W_4_R_5_], and [W_5_R_4_] [13]. The synthesis of [W_4_R_4_(DKP)] was achieved using Fmoc/tBu solid-phase peptide synthesis with a preloaded tryptophan 2-chlorotrityl resin (Fmoc-L-Trp(Boc)-2-chlorotrityl resin). The assembly of Fmoc-L-tryptophan and Fmoc-L-Arg(Pbf)-OH building blocks on the resin was followed by the coupling of Fmoc-Glu(OAll)-OH and Fmoc-Lys(Dde)-OH, respectively. Subsequently, selective deprotection of the allyl side chain of glutamic acid was accomplished in the presence of tetrakis(triphenylphosphine)palladium (0) and phenylsilane. The cyclization to DKP was then performed on the resin using 2-(1H-benzotriazol-1-yl)-1,1,3,3-tetramethyluronium hexafluorophosphate (HBTU) in *N*,*N*-diisopropylethylamine (DIPEA). Following the deprotection of Dde of lysine with hydrazine in *N*,*N*-dimethylformamide (DMF) (2%, *v*/*v*) and the cleavage of the resin in the presence of trifluoroethanol (TFE):acetic acid (AcOH):dichloromethane (DCM) (2:1:7 *v*/*v*/*v*), the side chain-protected peptide underwent cyclization in DMF using 1-hydroxy-7-azabenzotriazole (HOAt) and 1,3-diisopropylcarbodiimide (DIC). Finally, the deprotection step using reagent R containing trifluoroacetic acid/thioanisole/1,2-ethanedithiol (EDT)/anisole (90:5:3:2, *v*/*v*/*v*/*v*) yielded the dicyclopeptide [W_4_R_4_(DKP)] (Scheme 1).

### 2.3. Antibacterial Activity

The antibacterial activity of the synthesized peptides was evaluated against several Gram-positive and Gram-negative strains. The Gram-positive bacteria strains were methicillin-resistant *Staphylococcus aureus* (Los Angeles County (LAC) clone), *S. aureus* (ATCC 29213), *Enterococcus faecium* (ATCC 700221), *Enterococcus faecalis* (ATCC 29212), *Staphylococcus pneumoniae* (ATCC 51938), and *Bacillus subtilis* (ATCC 6633), while the Gram-negative bacteria strains were *Escherichia coli* (ATCC 25922), *Pseudomonas aeruginosa* (ATCC 27883), and *Klebsiella pneumoniae* (ATCC BAA 1705). Meropenem and daptomycin were used as the positive controls. [W_4_R_4_] was used for the comparative studies (Table 1). The minimum inhibitory concentration (MIC) values for all peptides were determined using a microbroth dilution assay.

Among all the peptides, [W_4_R_5_] was found to be the most potent in most of the strains. In comparison with [W_4_R_4_], as the number of W residues increased to five in [W_5_R_4_], the antimicrobial activities were reduced. For example, [W_5_R_4_] was four-fold, two-fold, and four-fold less potent than [W_4_R_4_] against *Klebsiella pneumoniae* (ATCC BAA 1705), *Pseudomonas aeruginosa* (ATCC 27883), and *E. coli* (ATCC 25922), respectively. In contrast, as the number of R residues increased to five in [W_4_R_5_], the antimicrobial activity was enhanced in most of the strains or remained the same. [W_4_R_5_] was two-fold and four-fold more potent than [W_4_R_4_] and [W_5_R_4_] against *P. aeruginosa* (ATCC 27883), respectively. Similarly, [W_5_R_4_] had two-fold higher activity than [W_4_R_4_] in *S. aureus* (ATCC 29213), *E. faecalis* (ATCC 29212), and *S. pneumoniae* (ATCC 51938). We reported previously that [W_4_R_5_] had about two-fold greater HC_50_ than [W_4_R_4_] (340 vs. 175) and had an SI (85.0) calculated for MRSA [13].

[W_4_R_4_(DKP)] demonstrated comparable activity with [W_4_R_4_] against *P. aeruginosa* (ATCC 27883) and *S. pneumoniae* (ATCC 51938) with MIC values of 64 and 4 µg/mL, respectively, while there was between 2 to 4-fold decrease in antibacterial activity in the other strains with the exception of *Bacillus subtilis* (ATCC 6633), which showed a dramatic reduction (64-fold) in activity. These data indicate that the insertion of a DKP ring or addition of another W residue into the cyclic peptide [W_4_R_4_] negatively affects its antimicrobial activity against most selected bacterial strains compared with the [W_4_R_4_] template, possibly due to reduced membranolytic activity by the inserted DKP in the amphiphilic peptide.

### 2.4. Molecular Dynamics Simulations

A series of all-atom MD simulations were conducted to investigate the specific peptide–membrane interactions that could potentially account for the diverse antimicrobial activities observed in a group of cyclic homologous peptides, namely, [W_4_R_4_], [W_5_R_4_], [W_4_R_5_], and [W_4_R_4_(DKP)]. These simulations were performed in both aqueous and water-membrane environments, aiming to provide a comprehensive understanding of the underlying mechanisms.

The spatial models of all the peptides clearly reveal their amphiphilic nature, with distinct regions on the surface exhibiting both hydrophilic and hydrophobic characteristics. The hydrophilic portion consists of positively charged arginines (R), while the hydrophobic part comprises tryptophan (W) residues (Figure 1 and Supporting Information, Figure S1).

#### 2.4.1. Conformational Behavior of Cyclic Peptides in Water

The spatial arrangement of peptides is primarily influenced by their cyclic structure. Throughout MD simulations in an aqueous solution, all peptides exhibit noticeable modifications of the initial “ring” model, resulting in the formation of backbone turns: approximately 90% of MD states for [W_5_R_4_] and about 50% for other peptides. In most of the accumulated MD states, one to two β-turns, defined according to the Ca_(*i*)_ − Ca_(*i* + 3)_ (*i* is the residue number) distance cutoff (<7 Å), were observed, frequently involving neighboring R and W residues. It is worth noting that even a single residue replacement in the 9-mer peptide leads to distinct distributions of possible β-turns for [W_4_R_5_] and [W_5_R_4_], regardless of whether the simulations were performed in water or the water–membrane environment (Supporting Information, Table S2).

The 8-mer peptide [W_4_R_4_] exhibited the least conformational mobility (in terms of RMSD values) in water, while the latter increased for the 9-mer peptides [W_4_R_5_] and [W_5_R_4_] (Table 2). The incorporation of an additional group (DKP-moiety) into the backbone in [W_4_R_4_(DKP)] not only enhanced the peptide flexibility but also resulted in the most pronounced structural reorganization compared with other peptides. To describe the propensity of the peptide to be structured in solution, hereinafter, we will use the term “plasticity”, which is defined as the average number of backbone H-bonds observed in the MD simulation of the peptide in solution (Table 2). In water, [W_5_R_4_] and [W_4_R_4_(DKP)] possess lower plasticity (the average number of H-bonds is 1.3) compared with more active peptides ([W_4_R_5_] and [W_4_R_4_]) with higher plasticity (0.7)).

The resulting clusters of MD conformers found in water were subsequently utilized in MD simulations in the presence of a membrane, as described in the Section 4 (Methods).

#### 2.4.2. Peptide–Membrane Interactions in the Water–Lipid Environment

The membrane binding of the peptides was studied with MD simulations in the presence of a two-component DOPC/DOPG lipid bilayer mimicking bacterial membrane. In all MD runs, the peptides exhibited tight binding to the lipid bilayer. While the peptides displayed flexibility in aqueous environments, their ability to adopt various conformations was notably restricted when interacting with the water-membrane medium.

##### Conformational Behavior of Membrane-Embedded Peptides

When the peptides were initially taken in conformations representing the two most prevalent clusters of states observed during MD simulations in water, their binding to the membrane did not induce significant changes in their spatial structure. The adaptation process primarily involved the adjustment of side chains to suit the water–lipid environment. The average RMSD values of the backbone atoms from the starting conformations remained below 1 Å. However, upon direct interaction between the peptides and the lipid bilayer, the “raw” starting models of the peptides underwent a rapid transformation into a conformational state, typically distinct from the initial “water” models. It should be noted that no convergence toward a single “bilayer-bound” state was observed for the peptide structures (Figure S2).

##### Membrane-Binding Modes

According to the MD data, the peptides exhibit behavior near the membrane that accurately replicates the primary range of membrane binding states previously observed in related charged amphiphilic peptides [22]. As a rule, two major membrane-binding modes and several intermediate states were elucidated using a series of independent MD simulations of the peptides in the hydrated bilayer.

**Figure 2:**
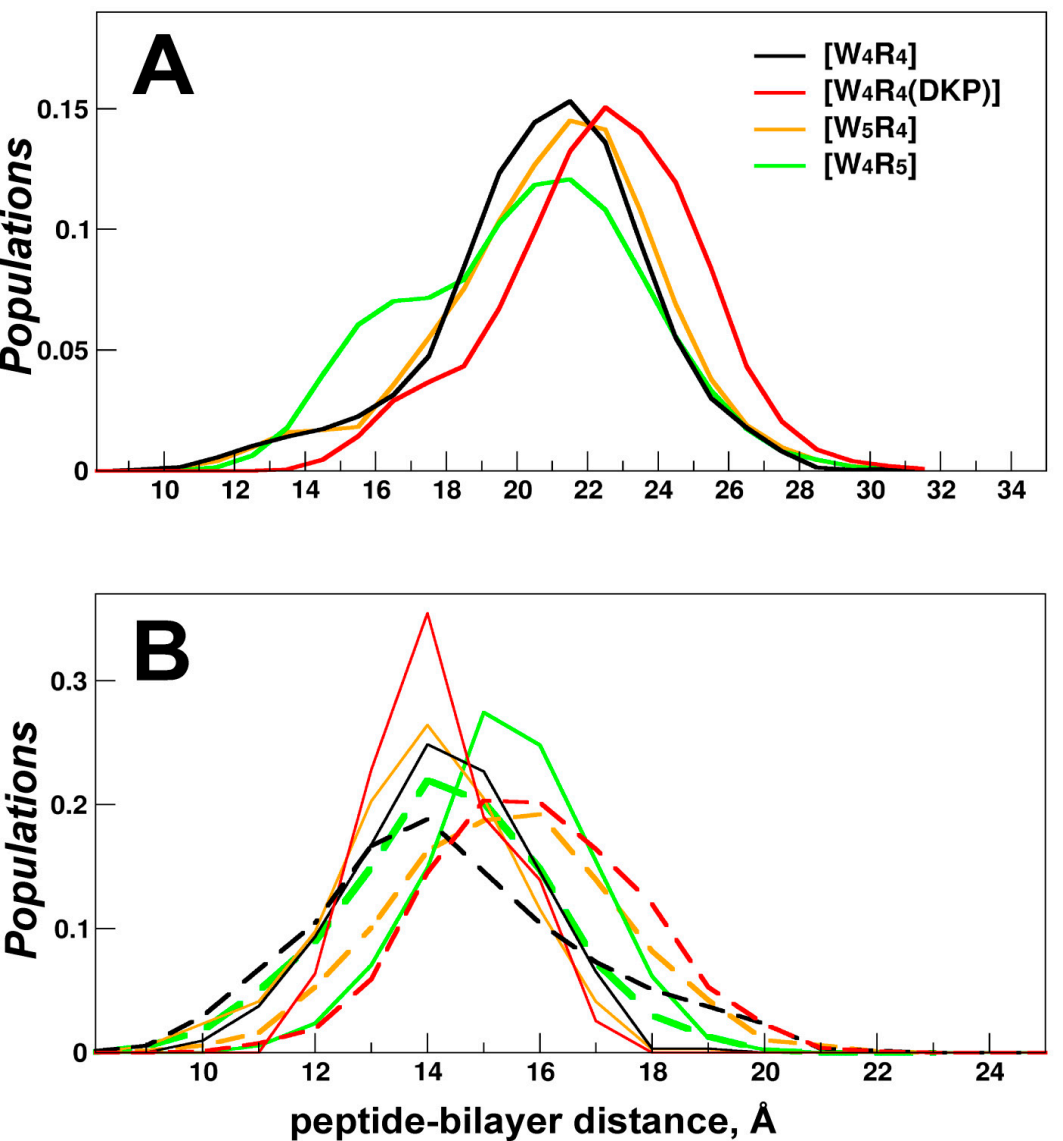
Depth of membrane insertion for cyclic peptides [W_4_R_4_], [W_5_R_4_], [W_4_R_5_], and [W_4_R_4_(DKP)]. The peptide indication color scheme (see the legend on panel **A**) is the same for both panels. (**A**) Distributions of the distances between the peptide center of mass and the bilayer center (“depth distribution”) were calculated over all MD states in 200–400 ns runs. (**B**) Comparison of depth distributions for MD states of peptides in the A-mode (observed in the initial MD time period (up to 400 ns)) and all MD states of µs-length MD runs (collected after the first 0.5 µs, dotted lines).

The electrostatically driven initial binding mode—the so-called “polar” mode (P-mode)—is highly populated. In this mode, charged side chains of the peptides insert into the lipid polar head region, thus making numerous favorable electrostatic contacts (including hydrogen bonds) with the membrane’s glycerophospholipids. In contrast, the hydrophobic Trp-motif remains either solvent-accessible or located at the polar water–lipid interface, and the center of mass (COM) of each Trp residue is above the P-atom line determined by averaging over all phosphate groups of the peptide-contacting lipid monolayer at each MD time step. As a result, the distance between the peptide center of mass and the center of the bilayer remained relatively large compared with other modes, as evident from the main peaks on the distributions illustrated in Figure 2A.

**Figure 3:**
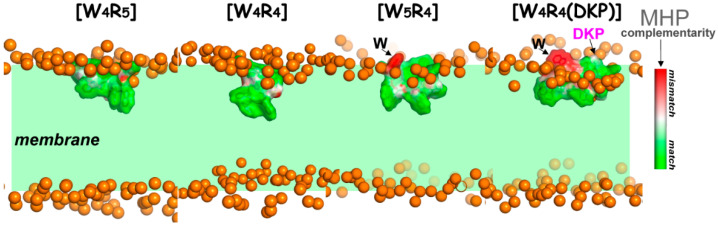
Complementarity of hydrophobic/hydrophilic surface properties for the membrane-bound states of the peptides [W_4_R_5_], [W_4_R_4_], [W_5_R_4_], and [W_4_R_4_(DKP)]. The molecular hydrophobicity potential (MHP) values induced with both peptide and water–lipid medium are calculated in the surface points of the peptide molecule. The color-coded scale for MHP match/mismatch values (peptide-induced to environment-induced) is given on the right. The phosphate groups of lipids are given as golden spheres. Water and lipid molecules are omitted for clarity, and the membrane region is indicated with a green background.

The functionally important membrane-active state of the peptide (named the *“apolar”* mode or A-mode) was recognized if all hydrophobic residues were embedded into the acyl chain region of the bilayer: the COM of each Trp residue was below the P-atom line. Thus, an amphiphilic peptide was inserted into the hydrophobic core of the membrane due to its apolar characteristics (Figure 3, the tryptophan motif inserted into the membrane). Consequently, the COM of the peptides (from the equilibrated part of µs-MD simulations) was closer to the membrane center by ca. 8 Å (Figure 2B). This second principal mode (A-mode) was observed much less frequently. Notably, all the peptides were also found in various intermediate states, indicating partial incorporation of their tryptophan motif into the membrane. In contrast with the *apolar* mode, in these intermediate states, the Trp residues were predominantly located in polar or weakly polar regions of the membrane.

**Figure 4:**
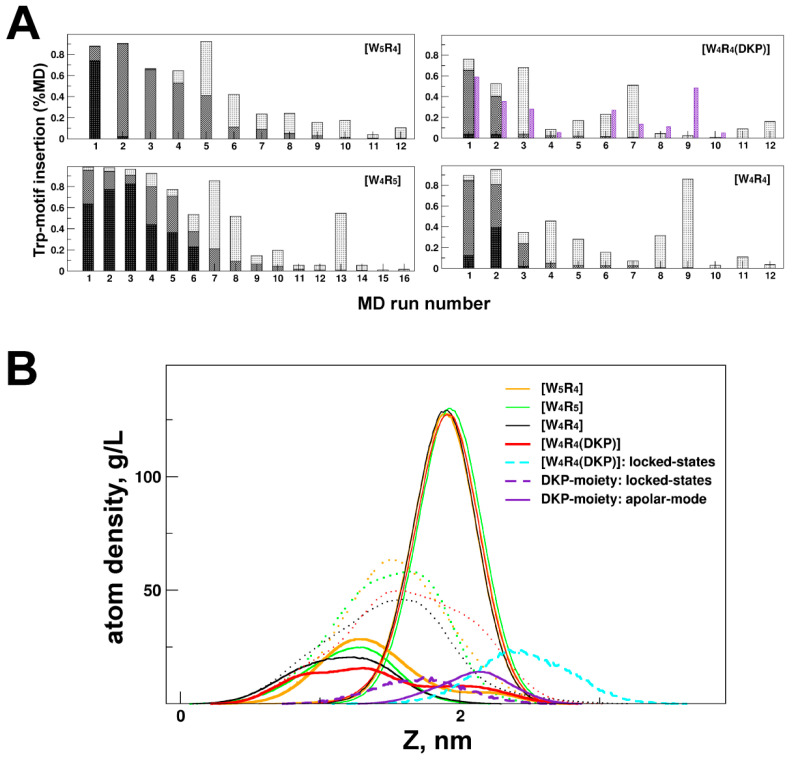
Membrane binding of four cyclic peptides via the tryptophan motif and/or DKP moiety. (**A**) *Apolar* and intermediate membrane binding modes (in % MD time) realized in each 200 ns MD run (a total of 12–16 for each peptide). The intensity of bin shading corresponds to a few of the incorporated tryptophan residues: at least one (light color), two (medium intensity), or all tryptophan motifs (dark, A-mode). The additional violet bins show a fraction of MD states with tightly membrane-bound DKP moiety of the [W_4_R_4_(DKP)] peptide. (**B**) Insertion depth of the peptides in A-mode as well as the [W_4_R_4_(DKP)] peptide in a locked state. The density distribution of phosphorous (P) atoms of lipids and Trp residues along the bilayer normal (Z) for the DOPC/DOPG membrane is shown. For the locked states of [W_4_R_4_(DKP)], localization of the W-motif and DKP atoms is indicated with cyan and violet dashed lines, respectively. MD-averaged (over the last 500 ns) atomic density distributions for the DKP moiety and the entire peptides in the A-mode are shown with a violet continuous line and dotted lines, respectively.

The occupancy of the A-mode (% of MD states) and the rate of embedding (the number of “successful” MD runs where the peptide binds to the membrane in the A-mode) were found to be highest for the most active [W_4_R_5_] and lowest for the least active [W_4_R_4_(DKP)] peptides (Figure 4A, Table 2). To gain further insights, several 200 ns-MD runs for the peptides [W_4_R_5_] and [W_4_R_4_] (which exhibited close antimicrobial activity) were extended up to 400 ns (see Table S1 for details). It was found that the peptides either remained incorporated within the membrane or displayed progress in embedding. Conversely, in three out of six extended MD runs conducted for the less active [W_5_R_4_], no progress in membrane binding (remaining at the intermediate stage) was observed.

To assess the stability of protein−membrane binding in the A-mode, two MD trajectories were further extended to 1 and 2 µs for [W_4_R_4_]/[W_4_R_4_ (DKP)] and 9-mer peptides ([W_5_R_4_]/[W_4_R_5_]), respectively. As depicted in Figure 2B, the depth of peptide insertion in A-mode, as observed in the nanosecond-range MD simulations (up to 400 ns), differs from that observed in the longer µs-MD simulations. In the latter case, the peak in the depth distribution was shifted toward a decrease in the embedding depth for the less active [W_5_R_4_] and, particularly, for the [W_4_R_4_(DKP)] peptide. Conversely, pronounced or minor progress in the binding depth was observed in µs-MD simulations for the more active [W_4_R_5_] and [W_4_R_4_] peptides, respectively.

The detection of a tendency for one residue from the tryptophan motif to remain more “float-up” rather than to be immersed was more apparent in long-term microsecond-scale MD simulations compared with nanosecond MD. This particular residue found itself in a non-complementary (non-favorable) environment, as indicated by the MHP data (Figure 3), exemplified by peptides [W_5_R_4_] and [W_4_R_4_(DKP)]. This non-complementary environment contributed to an increase in the energy required for peptide binding. Consequently, such behavior of the peptide, more pronounced for [W_5_R_4_] compared with [W_4_R_5_], where the tryptophan motif is smaller, led to a reduction in the overall binding depth of the peptide. On average, in the equilibrated membrane binding mode (after 0.5 μs), the peptides [W_4_R_5_] and [W_4_R_4_] exhibited deeper penetration with their hydrophobic region compared with [W_5_R_4_] and, particularly, [W_4_R_4_(DKP)] (Table 2).

##### Apolar Mode and Locked State of the DKP-Containing Peptide

For the [W_4_R_4_(DKP)] peptide, at least half of the simulations exhibited the insertion of the DKP moiety into the polar head group region of the membrane (Figure 4A, violet bins; Figure 4B, depth of insertion for the DKP-moiety, violet curve). Interestingly, even in MD runs with A-states and a high population of intermediate states (Figure 4A, start 1–3), a significant number of MD conformations revealed inserted DKP atoms. It is worth noting that the DKP moiety possesses amphiphilic properties, with a predominantly polar ring and a hydrophobic hydrocarbon chain (Figure 1). This allowed it to localize at the interface of polar/nonpolar regions in the bilayer. Such an energetically favorable disposition contributes to its stabilization in the membrane. This could be observed, for instance, in one of the MD trajectories of [W_4_R_4_(DKP)] (start 9 in Figure 4B), where the position of the DKP density peak was shifted toward the less polar region, closer to the membrane center (see Figure 4B). Consequently, according to the 400 ns-MD simulation data (start 9), the hydrophobic pattern of the peptide became firmly “trapped” in a peripheral position relative to the bilayer. In our view, these intermediate states referred to as the “locked mode” (Supporting Information, Figure S3), may contribute to a decrease in the membrane activity of the peptide.

The analysis of the MD data reveals that the incorporation of peptides into the membrane (A-mode) is possible from different initial conformational states of the molecule. At least two starting models of each peptide were able to integrate into the membrane with their hydrophobic pattern. At the same time, regardless of the starting structure, the key parameters governing the interactions of the peptides with the bilayer in the A-mode were similar. The contact area was approximately 70–80% of the peptide’s surface accessible to the solvent. Additionally, the peptide formed numerous (>10) intermolecular hydrogen bonds and established favorable electrostatic and hydrophobic contacts with the corresponding functional groups of lipids.

## 3. Discussion

The findings from the antimicrobial activity tests and molecular modeling data yield several conclusions about the relationship between structure and activity for the group of four cyclic peptides that were studied. It is important to note that these peptides have very small structural differences, with three of them being modifications of the original peptide [W_4_R_4_], each with the addition of one W or R residue or a residue containing a DKP group that exhibits “intermediate” hydrophobic properties between W and R. By comparing these derivatives to the parent peptide, it was observed that [W_4_R_5_] showed increased antimicrobial activity, while [W_5_R_4_] and [W_4_R_4_(DKP)] displayed decreased activity. Using an analysis of their structural–dynamic and hydrophobic properties, as well as their ability to interact with the model bacterial membrane, key SARs were identified.

Utilizing all-atom MD simulations, we validate the hypothesis that the enhanced antimicrobial activity of the peptide is linked to its capability to penetrate deeper into the membrane, ultimately reaching the hydrophobic core. Moreover, such a peptide demonstrates a large population of these MD-states, indicating greater thermodynamic stability in this binding mode. The latter proposal is supported by multiple independent MD runs, during which the peptide successfully penetrated the bilayer. The process of protein embedding encompasses numerous intermediate states including those resulting from the hydrophobic mismatch between the protein and the membrane, as well as energetically favorable interactions between charged lipids and arginine residues. The degree of hydrophobic insertion of the peptide becomes less as the energy of the transition states increases.

Among the studied peptides, the most active [W_4_R_5_] consistently achieves the successful incorporation of its entire hydrophobic pattern into the membrane across a greater number of independent MD runs than other peptides. The analysis of the membrane-bound states of the less active peptides [W_5_R_4_] and [W_4_R_4_(DKP)] within the initial nanosecond timeframe (200–400 ns) and in the more equilibrated microsecond range (after 0.5 µs) indicates a more pronounced inclination toward instability in the apolar mode. The instability contributes to a decrease in the depth at which these peptides are inserted into the membrane.

Due to the structural constraints of cyclic peptides, their conformational possibilities for distinguishing polar and nonpolar regions are limited. The larger the hydrophobic pattern, the more the backbone of Trp-motif bends and the higher the probability of simultaneous embedding of charged Arg residues. Consequently, this leads to energetically unfavorable interactions of polar/charged groups (backbone atoms and Arg side chains) embedded into the fatty acyl chain region of the membrane. As a result, the equilibrium distribution of membrane-bound states shifts toward less buried states. The “breathing” behavior of the peptide, where it alternates between sinking and floating up within the equilibrated segments of long-term MD trajectories is observed. As depicted in Figure 2B and Figure 4B, the distribution of insertion depths for all peptides remains broad.

Based on the MD data, we assume that an increase in the size of the hydrophobic motif ([W_5_R_4_] compared with [W_4_R_5_]) restricts its ability to penetrate deeply into the membrane. This limitation arises from the simultaneous insertion of functional peptide groups with physicochemical properties that are not complementary to those of the water/lipid environment. Indeed, a shift in amphipathy toward an increase in the size of the hydrophobic pattern (addition of W versus R) while maintaining the total number of residues in the cyclic peptide leads to a reduction in antimicrobial activity as accentuated with larger cyclic peptides. The larger the size of the apolar pattern, the more significant this dependence becomes [13]. A similar trend was observed in peptides containing the same number of R residues but varying numbers of W residues. For instance, comparing [W_7_R_3_] to [W_4_R_3_]; [W_7_R_4_] to [W_5_R_4_] and [W_4_R_4_]; [W_7_R_5_] to [W_4_R_5_]; and [W_7_R_6_] to [W_4_R_6_], peptides with a higher number of W residues exhibited reduced antimicrobial activity. The observed structure–functional patterns also do not exclude the next assumption. Deep penetration of the continuous hydrophobic pattern into the hydrophobic core may not be advantageous or less effective for their antimicrobial function, likely due to stronger hydrophobic interactions and the consequent generation of membrane stability.

Furthermore, we found a correlation between the decrease in the plasticity of the cyclic structure (attributed to the formation of turns and their stabilization by intramolecular hydrogen bonds) and a decrease in antimicrobial activity ([W_5_R_4_], [W_4_R_4_(DKP)] versus [W_4_R_5_] and [W_4_R_4_]; see Table 2). Considering the sharp decline in the conformational capabilities of the peptides observed during MD simulations when interacting with the membrane, an augmentation in the backbone rigidity further diminishes the ability of the peptide to adapt to the lipid environment.

The introduction of a DKP group, which is a flexible moiety with amphiphilic properties, into a cyclic structure ([W_4_R_4_(DKP)] peptide) results in the emergence of unproductive modes of membrane binding. The DKP group tends to localize at the interface of polar and nonpolar zones of the bilayer. As a result, the hydrophobic pattern of the peptide can either become “trapped” near the bilayer surface (Supporting Information, Figure S3) or be inserted less deeply (Figure 3).

A comprehensive summary of the obtained results can be clearly illustrated using the SAR profiles for each peptide, which are strings of characters denoting specific features (designated as S1–S8 in Table 3). The numerical value of each of them can be either 1 (represented by the symbol “●” in Table 3) or 0 (represented by the symbol “○”). These features are determined based on the peptide’s “Activity” (S1–S5) and its effectiveness in embedding into the non-polar region of the bilayer (described in the “Modeling” section of Table 3, S6–S8).

For the peptide where these parameters reach their extreme values, the highest antimicrobial (S1–S4) and lowest hemolytic (S5) activities, as well as the maximum values of MD-elucidated features (S6–S8), the respective signs are set to 1. The MD-related features were evaluated by comparing the four studied peptides, while activity parameters were determined using four additional analogs with an increased content of W and R residues (Table 3).

It is evident that the peptide [W_4_R_5_] possesses the optimal pattern in terms of “activity/binding to the bacterial membrane” and exhibits the best characteristics in both sections of the profile—the “experimental” (S1–S5) and the “calculated” ones (S6–S8). Among others, the peptide [W_4_R_5_] demonstrated the most favorable HC_50_ value of 340 µg/mL and displayed low MIC values of 4 µg/mL, 32 µg/mL, 32 µg/mL, and 16 µg/mL against MRSA (ATCC BAA-1556), *K. pneumoniae* (ATCC BAA-1705), *P. aeruginosa* (ATCC 27883), and *E. coli* (ATCC 25922), respectively. A very similar SAR profile (most of the features equal to “1”) was found for [W_4_R_4_], which shows almost identical antimicrobial activity but has a lower SI value (43.8 instead of 85 for [W_4_R_5_]) due to being more hemolytically active.

Comparing [W_4_R_4_] with the peptides [W_5_R_4_], [W_6_R_4_], and [W_7_R_4_], which have the same number of R residues and an increased number of W residues, differences in both the SAR profile and the activity of these peptides become pronounced [13]. As the number of W residues increased (from four to seven), a significant loss of antimicrobial activity of these peptides was observed, showing MIC values of 4 µg/mL, 8 µg/mL, 128 µg/mL, and 256 µg/mL against MRSA (ATCC BAA-1556), respectively. Also, a noticeable decrease in the HC_50_ values was demonstrated (from 175 to 60 µg/mL) in this set of peptides.

Considering the previous information regarding the impact of adding W and R residues to the parent peptide [W_4_R_4_], it is reasonable to assume that the introduction of another R into the latter will enhance the interaction between the positively charged guanidine groups of arginine residues and the negatively charged groups of polar lipid heads and with water molecules, making it unprofitable to embed the peptide deeply into the membrane. The predominance of R residues over W residues in a cyclic peptide impairs the activity profile, although not so clearly. Thus, [W_4_R_6_] and [W_4_R_7_] were generally less active than [W_4_R_5_] and [W_4_R_4_] (SI values of 33 and 12 versus 85 and 43.8) [13].

Based on the correlation between the MD results and the activity of the four “basic” peptides, it is possible to extrapolate the SAR profiles to the 10-/11-residue peptides listed in Table 3, which only have activity indicators available. In summary, these 10/11-residue analogs do not exhibit both high antimicrobial activity and low hemolytic activity compared with the most promising peptide [W_4_R_5_].

The SAR model showed a strong correlation with our previous experimental results [13]. Comparatively, [W_4_R_6_] and [W_4_R_7_], which had a similar number of W residues but a higher number of R residues, exhibited lower HC_50_ values when compared with [W_4_R_5_]. [W_4_R_6_] was generally less active than [W_4_R_5_] against three bacterial strains, MRSA (ATCC BAA-1556), *K. pneumoniae* (ATCC BAA-1705), *P. aeruginosa* (ATCC 27883), and *E. coli* (ATCC 25922). [W_4_R_7_] was also less active than [W_4_R_6_] against these bacteria. When comparing [W_4_R_4_] with the peptides [W_5_R_4_], [W_6_R_4_], and [W_7_R_4_], which had similar numbers of R residues and different numbers of W residues, the differences became more pronounced. Thus, the addition of W residues led to a noticeable increase in hemolytic activity and a significant decrease in antimicrobial activity. In general, the order of antimicrobial activity was observed as follows: [W_4_R_4_] > [W_5_R_4_] > [W_6_R_4_] > [W_7_R_4_], which is reflected in the SAR profiles (Table 3).

## 4. Materials and Methods

### 4.1. Materials

Fmoc-L-Trp(Boc)-2-chlorotrityl resin (loading 0.37 mmol/g) was purchased from Sigma-Aldrich (St. Louis, MO, USA). Protected Amino acids were purchased from AAPPTec LLC (Louisville, KY, USA). TFA, phenylsilane, acetic acid, DMF, HBTU, tetrakis(triphenylphosphine)palladium (0), thioanisole, EDT, DIPEA, hydrazine hydrate, piperidine, triisoproylsilane (TIS), HOAt, DIC, and all other reagents were bought from MilliporeSigma (St. Louis, MO, USA). All bacterial experiments were carried out under a laminar flow hood Labconco (Kansas City, MO, USA). All bacterial strains except MRSA (Los Angeles County (LAC) clone) used in this study were procured from the American Type Culture Collection (ATCC).

### 4.2. Chemical Synthesis of [W_4_R_4_(DKP)]

We previously reported the synthesis of [W_4_R_4_], [W_5_R_4_], and [W_4_R_5_] [13]. The sequence W_4_R_4_ was assembled on resin using Fmoc/tBu solid-phase peptide synthesis using tryptophan preloaded trityl resin (Fmoc-L-Trp(Boc)-2-chlorotrityl resin, 0.37 mmol/g, 0.3 mmol, 810 mg) as previously reported [13]. A solution of Fmoc-Glu(OAll)-OH (369 mg), HBTU (341 mg), and DIPEA (315 µL) in DMF (20.0 mL) was added to the resin. The reaction was continued under nitrogen for 2 h, followed by draining of the solution and washing with DMF (2 × 10.0 mL). A solution of 20% piperidine in DMF (20 mL, *v*/*v*) was added to remove N-terminal Fmoc. The reaction was continued for 20 min followed by washing with DMF (2 × 10.0 mL). The deprotection with 20% piperidine in DMF (*v*/*v*) and washing with DMF (2 × 10.0 mL) was repeated. A solution of Fmoc-Lys(Dde)-OH (480 mg), HBTU (341 mg), and DIPEA (315 µL) in DMF (20.0 mL) was added to the resin. The reaction was continued under nitrogen for 2 h, followed by draining the solution and washing with DMF (2 × 10.0 mL). A solution of 20% piperidine in DMF (20.0 mL, *v*/*v*) was added, and the reaction was continued for 20 min followed by washing with DMF (2 × 10.0 mL). Then, another addition of 20% piperidine in DMF (*v*/*v*) was repeated, followed by washing with DMF (2 × 10.0 mL) to deprotect the Fmoc of lysine. The resin was washed with anhydrous DCM (3 × 10 mL). Tetrakis(triphenylphosphine)palladium catalyst (0.1 equiv., 34.7 mg) was dissolved in 10 mL DCM, and then 20 equiv. of phenylsilane (740 µL) was added. The whole mixture was added to the resin. Agitation was performed for 30 min under nitrogen. The same previous steps were repeated again followed by washing with DCM (3 × 10 mL) to remove the allyl group of the glutamic acid. A solution of HBTU (341 mg) and DIPEA (315 µL) in DMF (20.0 mL) was added to the resin to accomplish the cyclization. The reaction was continued under nitrogen for 2 h. It was followed by draining the solution and washing with DMF (5 × 10.0 mL). A solution of 2% hydrazine was prepared by dissolving 3.0 mL of 98% solution of hydrazine monohydrate in 103.4 mL of DMF. The solution (10 mL) was added to the resin and agitated for 15 min, followed by washing with DMF (3 × 10 mL). The same previous steps were repeated once to remove the Dde of lysine. The side chain-protected peptide was cleaved from the resin using a solution mixture of trifluoroethanol (TFE)/acetic acid/dichloromethane (DCM) (20 mL, 2:1:7 *v*/*v*/*v*) for 2 h to cleave the resin. The resin was filtered off, and the liquid was evaporated to dryness to obtain a side-chain-protected linear peptide. The side chain-protected peptide was taken in a 500 mL round bottom flask under nitrogen. DMF (200 mL) and DCM (50 mL) were added to the protected peptide. A solution of HOAt (136 mg) in 4 mL DMF and 208 µL of DIC were added to cyclize the peptide. The reaction was stirred for 18 h under nitrogen. The solvents were evaporated under a vacuum. Removal of functional protecting groups was performed by stirring the peptide in a 15 mL solution of TFA/thioanisole/EDT/anisole (90:5:3:2 *v*/*v*/*v*/*v*) for 2 h. After filtration of the resin, cold diethyl ether (15.0 mL) was added to the filtrate to precipitate the peptide that was purified with RP-HPLC (Shimadzu LC-20AP), using a gradient system from 0 to 100% acetonitrile (CH_3_CN) and water containing 0.1% (*v*/*v*) TFA using a preparative C18 column (Gemini, 5 μm particle size, 100 Å pore size, 21.2 mm × 250 mm) from Phenomenex. The peptide had an HPLC purity of >95%. MALDI-TOF (*m*/*z*): C_79_H_105_N_27_O_11_: calculated, 1608.9168; found, 1609.3731 [M + H]^+^.

### 4.3. Antibacterial Assay

The selected strains of bacteria were cultured according to the guidelines of the Clinical Laboratory Standards Institute (CLSI) to determine MIC values using a microbroth dilution assay. The MIC values were determined to be at concentrations in wells in which no visible bacterial growth was present. An aliquot of an overnight culture of bacteria was grown according to the guidelines of CLSI, diluted in 1 mL normal saline to achieve 0.5 McFarland turbidity (1.5 × 10^8^ bacterial cell CFU/mL). Then, 60 µL of the 0.5 McFarland solution was added to 8940 µL of MH media (a 1/150 dilution). The tested peptides (256 µg/mL) were prepared from a stock solution of the samples for testing in Mueller Hinton Broth MH media. An amount of 100 µL MH media was pipetted into the sterile 96-well plate except for the first well. An amount of 200 µL of the 256 µg/mL compound samples was added by pipette into the first well and serially diluted with the MH media along sterile 96 wells using a multi-channel pipette except the last well. An amount of 100 µL aliquot of bacteria solution was added to each well, and the plate was incubated at 37 °C for 24 h. All experiments were conducted in triplicate.

### 4.4. Molecular Dynamics Simulations

To study the peptide–membrane interactions, a series of MD simulations of the peptides [W_4_R_4_], [W_5_R_4_], [W_4_R_5_], and [W_4_R_4_(DKP)] was conducted in both water and a hydrated pre-equilibrated negatively charged lipid bilayers. The latter was composed of DOPC and DOPG lipids, and their ratio (7:3) was chosen to match our previous experiments (16) and accurately represented the lipid composition of the bacterial plasma membrane. Specifically, the two-component bilayer consisted of 96 DOPC and 32 DOPG molecules.

#### 4.4.1. Preparation of the Starting Configurations

The peptides’ initial spatial arrangements were constructed using the molecular builder Avogadro version 1.2.0 [23] (https://avogadro.cc/, accessed on 9 December 2023) To probe the conformational mobility in water, the peptides were subjected to simulation in an aqueous solution at 310 K. For all peptides, three independent simulations were performed, thus giving a total MD time of 600 ns in each case. The preparation and production stages of the MD process were consistent for all peptides and were described previously [22].

Two representatives of the most populated clusters (root-mean-square deviation (RMSD)-based clustering algorithm) that dominated in water simulations were selected as initial configurations for conducting MD simulations of each peptide in the water–bilayer environment. To clarify how the initial protein conformation affects the resulting mode of peptide–membrane binding, we performed a series of 200 ns MD calculations with three starting models for each peptide. Two models were taken from MD data in water and the third one was the original model (close to the structure of “ideal ring”) whose energy was only minimized in water.

A minimum of 12 (16 for [W_4_R_5_]) independent MD runs (4 for each starting model) were carried out in the DOPC/DOPG bilayer. In all MD starts, the peptides were initially fully exposed to water and positioned adjacent to the membrane surface. A complete description of MD trajectories (number, length, starting structures, etc.) can be seen in Table S1. For the peptides with a membrane-embedded hydrophobic pattern (Trp-motif), the corresponding MD runs (see Table S1 for details) were extended to 400 ns. Additionally, for each peptide, at least two MD trajectories were further prolonged to 1 μs for 8-mer peptides or 2 μs for 9-mer peptides. This was completed to assess the stability of the peptide–membrane binding mode when the Trp-motif was embedded into the membrane. Overall, the total simulation times were 7.6 µs, 6.8 µs, 4.2 µs, and 4.4 µs for the peptides [W_4_R_5_], [W_5_R_4_], [W_4_R_4_], and [W_4_R_4_(DKP)], respectively.

#### 4.4.2. MD Protocols

MD simulations were performed using the GROMACS **[24]** package version 2020.4 and the all-atom CHARMM36 [25] force field. Molecular topology for the peptide [W_4_R_4_(DKP)] was generated using the set of CHARMM36 force field parameters (bonded and nonbonded) for proteins [26]. In all calculations, the tip3p [27] water model and 3D periodic boundary conditions were used. After peptide insertion, the peptide–bilayer systems were resolvated (ca. 5.500 water molecules) within boxes ca. 66 × 66 × 77 Å^3^ in size. To keep the system electrically neutral, Na^+^ counterions were added. A spherical cutoff function (12 Å) and particle mesh Ewald (PME) algorithm [28] (with a 12 Å cutoff) were used to treat van der Waals and electrostatic interactions, respectively. The preparation and production MD stages were the same for all peptides and described elsewhere [22]. Finally, MD production runs (at least 200 ns each) were conducted in an NPT ensemble at a semi-isotropic pressure and a constant temperature of 310 K with an integration time step of 2 fs.

#### 4.4.3. Data Analysis

MD trajectories were sampled for analysis at time intervals of 100–1000 ps. MD data were analyzed and averaged over the accumulated sets of MD trajectories. The conformational mobility of the peptides was evaluated using GROMACS utilities (*gmx rmsd*, *gmx cluster*). Lipid (phosphorus atoms) and peptide density profiles were obtained using *gmx density* utility. Intermolecular interactions (electrostatic, hydrophobic contacts, and hydrogen bonds) and secondary structures, as well as the depth of peptide insertion into the membrane, were evaluated using GROMACS tools (*gmx distance*, *gmx hbond*) and original in-house software utilities. The geometric criteria used to detect electrostatic and hydrophobic intermolecular interactions are described elsewhere [22]. The total and non-polar accessible surface areas of the peptides were estimated using *naccess* v. 2.1.1 software [29].

The distribution of hydrophobic/hydrophilic properties on the molecular surfaces of the peptides was calculated using the molecular hydrophobicity potential (MHP) approach as described previously [22]. For the analysis, MHP values were calculated in log *P* units, where *P* is the octanol−water distribution coefficient.

Mapping the hydrophobic properties of the peptides’ surfaces (Figure S1) was performed using the Protein Surface Topography technique [30], and its complementarities to the water–membrane environment were estimated with the freely available Platinum v. 1.0 software as described previously [22]. Molecular graphics were rendered using PyMOL v. 2.5 (http://pymol.org/, accessed on 9 December 2023).

## 5. Conclusions

The cyclization of peptides clearly plays a significant role in maintaining a stable “amphiphilic portrait”, which is crucial for direct membrane activity and the antimicrobial potential of the peptides. In contrast to linear molecules, cyclic peptides offer enhanced opportunities for designing and controlling these physicochemical properties more effectively. For the designed cyclic peptides ([W_4_R_4_], [W_5_R_4_], [W_4_R_5_], and [W_4_R_4_(DKP)]), both the comparative antimicrobial evaluation and MD simulations within the model bilayer mimicking the bacterial membrane were conducted. [W_4_R_5_] was found to be the most potent among the selected peptides. According to the proposed SAR profile, this peptide has an optimal “activity/binding to the bacterial membrane” pattern. Extension of the hydrophobic pattern (with the incorporation of the Trp residue), as well as introducing the amphiphilic DKP-fragment into the parent peptide [W_4_R_4_] with the same content of W and R residues, does not lead to an increase in the antimicrobial potential of the corresponding peptides. Overall, the final parameters of the distribution of the membrane binding modes were influenced by a complex interplay of factors such as the plasticity/rigidity of cyclic structure, the size of the hydrophobic pattern, and the geometry of binding. The main message conveyed by this study is that achieving such finely regulated SAR profiles for a series of homologous peptides with different activities can only be deciphered using a combination of chemical synthesis, biological testing, and molecular modeling techniques.

## Figures and Tables

**Table 1:** Antibacterial activity of [W_4_R_4_], [W_4_R_5_], [W_5_R_4_], and [W_4_R_4_(DKP)] against bacterial pathogens.

					MIC (µg/mL) ^a^	
	[W_4_R_4_]	[W_4_R_5_]	[W_5_R_4_]	[W_4_R_4_(DKP)]	Meropenem	Daptomycin
MRSA (ATCC BAA-1556) (LAC clone)	4	4	8	32	2	ND
*Klebsiella pneumoniae* (ATCC BAA 1705)	32	32	128	64	16	ND
*Pseudomonas aeruginosa* (ATCC 27883)	64	32	128	64	1	ND
*E. coli* (ATCC 25922)	16	16	64	32	1	ND
*S. aureus* (ATCC 29213)	8	4	ND ^b^	32	ND	1
*E. faecium* (ATCC 700221)	4	4	ND	16	ND	2
*E. faecalis* (ATCC 29212)	16	8	ND	64	ND	16
*S. pneumoniae* (ATCC 51938)	4	2	ND	4	ND	8
*Bacillus subitilis* (ATCC 6633)	1	4	ND	64	ND	0.5

^a^ Results of three independent experiments performed in triplicate; ^b^ ND = not determined.

**Table 2:** Main parameters of membrane-bound states (A-mode) and conformational mobility in water for a set of cyclic peptides with different antimicrobial activities.

A-Mode ^a^:	[W_4_R_5_]	[W_4_R_4_]	[W_5_R_4_]	[W_4_R_4_(DKP)]
Number of 200 ns-MD runs	6 from 16	3 from 12	2 from 12	2 from 12
%MD states in 200 ns/400 ns-MD runs	20.4/30.0	6.7/11.0	6.3/10.8	0.8/2.8
Binding depth in µs-MD (Å) ^b^	15.2 ± 1.8/ 12.9 ± 1.9	14.9 ± 2.4/ 11.9 ± 2.2	15.8 ± 2.0/ 14.2 ± 2.0	16.4 ± 1.8/ 15.2 ± 2.1
Stability of deep membrane binding in µs-MD ^c^	+	+	−	−
**Conformational behavior in water:**				
RMSD, Å ^d^	1.7 ± 0.5	0.9 ± 0.3	1.6 ± 0.7	2.1 ± 0.8
Backbone plasticity: number of h-bonds ^e^	0.7 ± 0.8	0.7 ± 0.7	1.3 ± 1.0	1.3 ± 0.8

^a^ Potential membrane-active state of peptides, in which all hydrophobic residues (Trp-motif) reach the apolar core of the membrane (see the Section 2 (Results) for details). ^b^ Estimated as the average distance between the bilayer center and the center of mass (COM) of entire peptide molecule/COM of the hydrophobic Trp-motif of the peptide. These values were calculated over all µs-MD runs (after the first 0.5 µs). ^c^ Based on the depth distributions (see Figure 2B) calculated only for the A-mode population observed in MD trajectories of 200–400 ns length and for all MD states of the corresponding µs-term MD (after 0.5 µs). Stable (+) or non-stable (−) membrane binding means no shift in the depth distribution toward the water solution or a decrease in the binding depth, respectively. ^d^ Root-mean-square deviation (RMSD) values of the backbone atoms’ coordinates were calculated between all pairs of MD conformers and averaged. ^e^ Average number of intramolecular backbone H-bonds observed in MD simulations in water.

**Table 3:** Structure–activity relationships for W/R-consisting cyclic peptides with varied numbers of W/R residues.

	Peptide	SAR-Profile							
		Activity ^a^					Modeling ^b^		
		S1	S2	S3	S4	S5	S6	S7	S8
		MRSA	*K. pneumoniae*	*P. aeruginosa*	*E. coli*	HC_50_	Backbone plasticity	Binding Depth	Inserted W-motif
	[W_4_R_7_]	○ ^c^	○	○	○	○			
+R	[W_4_R_6_]	○	○	●	○	○			
↑ ^d^	[W_4_R_5_]	●	●	○	●	●	●	●	●
	[W_4_R_4_]	●	●	○	●	○	●	●	○
↓	[W_5_R_4_]	○	○	○	○	○	○	○	○
+W	[W_6_R_4_]	○	○	○	○	○			
	[W_7_R_4_]	○	○	○	○	○			
	[W_4_R_4_(DKP)]	○	○	○	○		○	○	○

^a^ Activities are evaluated based on the values of MIC (µg/mL) for MRSA (LAC clone) (S1), *Klebsiella pneumoniae* (ATCC BAA 1705) (S2), *Pseudomonas aeruginosa* (ATCC 27883) (S3), *E. coli* (ATCC 25922) (S4), and values of HC_50_ (S5) (data from [13]). HC_50_ is the concentration of a peptide in μg/mL at which 50% hemolysis was observed. ^b^ Based on MD-data (see Table 2) and indicates the level of conformational plasticity of the peptide (S6), the depth of its immersion in the membrane (S7), and the population (%MD states) of A-mode of the membrane binding (S8). ^c^ The peptide that showed the extremal value of a given property (S1–S4, S6–S8—see text for details) among the other peptides from the list (column on the left) is marked with the symbol “●”; otherwise, it is marked with “○”. For hemolytic activity (S5), the minimum HC_50_ value is indicated with the symbol “●”. The SAR profile for the “parent” peptide [W_4_R_4_] is grey-hatched. ^d^ Up and down arrows indicate an increase in the number of Arg and Trp residues, respectively, in the corresponding peptides (on the right).

## Data Availability

Publicly available datasets were analyzed in this study. This data can be found here: [https://zenodo.org/record/8195619] accessed on 9 December 2023. This archive contains the initial structures of peptides used for modeling their interaction with the membrane. It includes the molecular topologies of these cyclic peptides and important parameters used for successful MD (molecular dynamics) runs. Additionally, representative MD trajectories of peptide–membrane interactions, accompanied by corresponding coordinate files in .gro and .pdb formats are also included. These MD simulations vividly demonstrate the peptide binding process to the DOPC/DOPG bilayer, along with key findings derived from analyzing the extensive MD data pool. The majority of the data analysis was performed using standard Gromacs utilities and freely available software, as specified in the Section 4 (Methods). Moreover, custom scripts and utilities are available upon request.

## Supplementary Materials

The following supporting information can be downloaded at: https://www.mdpi.com/article/10.3390/molecules28248049/s1, Table S1. Description of the obtained MD trajectories: number, length, starting structures, and the presence of functionally active membrane-bound states (*apolar* mode); Table S2. Structuring of the peptides in water and water–membrane environments as probed with MD simulations: occurrence (%MD) of β-turns; Figure S1. Distribution of hydrophobic/hydrophilic properties on the molecular surface of the peptides [W_4_R_4_], [W_5_R_4_], [W_4_R_5_], and [W_4_R_4_(DKP)]; Figure S2. µs-long MD simulations: different conformations of membrane-embedded states of the peptides [W_4_R_4_], [W_5_R_4_], [W_4_R_5_], and [W_4_R_4_(DKP)]; Figure S3. Two membrane binding modes (*apolar* and *locked*) of the peptide [W_4_R_4_(DKP)] are potentially important for its membrane activity.

## References

[1] Wang L., Wang N., Zhang W., Cheng X., Yan Z., Shao G., Wang X., Wang R., Fu C. (2022). Therapeutic peptides: Current applications and future directions. Signal Transduct. Target. Ther..

[2] Khairkhah N., Namvar A., Bolhassani A. (2023). Application of cell penetrating peptides as a Promising drug carrier to combat viral infections. Mol. Biotechnol..

[3] Freimann K., Arukuusk P., Kurrikoff K., Vasconcelos L.D.F., Veiman K.L., Uusna J., Margus H., Garcia-Sosa A.T., Pooga M., Langel Ü. (2016). Optimization of in vivo DNA delivery with NickFect peptide vectors. J. Control. Release.

[4] García-Sosa A.T., Tulp I., Langel K., Langel Ü. (2014). Peptide-ligand binding modeling of siRNA with cell-penetrating peptides. Biomed. Res. Int..

[5] Kang S.-J., Kim D.-H., Mishig-Ochir T., Lee B.-J. (2012). Antimicrobial peptides: Their physicochemical properties and therapeutic application. Arch. Pharmacal. Res..

[6] Avci F.G., Akbulut B.S., Ozkirimli E. (2018). Membrane active peptides and their biophysical characterization. Biomolecules.

[7] Fjell C.D., Hiss J.A., Hancock R.E., Schneider G. (2011). Designing antimicrobial peptides: Form follows function. Nat. Rev. Drug Discov..

[8] Azmi F., Skwarczynski M., Toth I. (2016). Towards the development of synthetic antibiotics: Designs inspired by natural antimicrobial peptides. Curr. Med. Chem..

[9] Falanga A., Nigro E., De Biasi M.G., Daniele A., Morelli G., Galdiero S., Scudiero O. (2017). Cyclic peptides as novel therapeutic microbicides: Engineering of human defensin mimetics. Molecules.

[10] Huang Y., Huang J., Chen Y. (2010). Alpha-helical cationic antimicrobial peptides: Relationships of structure and function. Protein Cell.

[11] Erdem Büyükkiraz M., Kesmen Z. (2022). Antimicrobial peptides (AMPs): A promising class of antimicrobial compounds. J. Appl. Microbiol..

[12] Jindal M., Le C., Mohd Yusof M., Sekaran S. (2014). Net charge, hydrophobicity and specific amino acids contribute to the activity of antimicrobial peptides. J. Health Transl. Med..

[13] Mohammed E.H.M., Lohan S., Ghaffari T., Gupta S., Tiwari R.K., Parang K. (2022). Membrane-active cyclic amphiphilic peptides: Broad-spectrum antibacterial activity alone and in combination with antibiotics. J. Med. Chem..

[14] Sammes P.G. (1975). Naturally occurring 2,5-dioxopiperazines and related compounds. Fortschr. Chem. Org. Naturst..

[15] De Rosa S., Mitova M., Tommonaro G. (2003). Marine bacteria associated with sponge as source of cyclic peptides. Biomol. Eng..

[16] Bugni T.S., Ireland C.M. (2004). Marine-derived fungi: A chemically and biologically diverse group of microorganisms. Nat. Prod. Rep..

[17] Huang R., Zhou X., Xu T., Yang X., Liu Y. (2010). Diketopiperazines from marine organisms. Chem. Biodivers..

[18] Abderhalden E., Komm E. (1924). The formation of diketopiperazines from polypeptides under various conditions. Z. Physiol. Chem..

[19] De Carvalho M.P., Abraham W.-R. (2012). Antimicrobial and biofilm inhibiting diketopiperazines. Curr. Med. Chem..

[20] Martins M.B., Carvalho I. (2007). Diketopiperazines: Biological activity and synthesis. Tetrahedron.

[21] Bojarska J., Mieczkowski A., Ziora Z.M., Skwarczynski M., Toth I., Shalash A.O., Parang K., El-Mowafi S.A., Mohammed EH M., Elnagdy S. (2021). Cyclic Dipeptides: The biological and structural landscape with special focus on the anti-cancer proline-based scaffold. Biomolecules.

[22] Lohan S., Mandal D., Choi W., Konshina A.G., Tiwari R.K., Efremov R.G., Maslennikov I., Parang K. (2022). Small amphiphilic peptides: Activity against a broad range of drug-resistant bacteria and structural insight into membranolytic properties. J. Med. Chem..

[23] Hanwell M.D., Curtis D.E., Lonie D.C., Vandermeersch T., Zurek E., Hutchison G.R. (2012). Avogadro: An advanced semantic chemical editor, visualization, and analysis platform. J. Cheminform..

[24] Abraham M.J., Murtola T., Schulz R., Páll S., Smith J.C., Hess B., Lindahl E. (2015). GROMACS: High performance molecular simulations through multi-level parallelism from laptops to supercomputers. SoftwareX.

[25] Klauda J.B., Venable R.M., Freites J.A., O’Connor J.W., Tobias D.J., Mondragon-Ramirez C., Vorobyov I., MacKerell Jr A.D., Pastor R.W. (2010). Update of the CHARMM all-atom additive force field for lipids: Validation on six lipid types. J. Phys. Chem. B.

[26] Best R.B., Zhu X., Shim J., Lopes P.E., Mittal J., Feig M., MacKerell A.D. (2012). Optimization of the additive CHARMM all-atom protein force field targeting improved sampling of the backbone ϕ, ψ and side-chain χ1 and χ2 dihedral angles. J. Chem. Theory Comput..

[27] Jorgensen W.L., Tirado-Rives J. (2005). Potential energy functions for atomic-level simulations of water and organic and biomolecular systems. Proc. Natl. Acad. Sci. USA.

[28] Essmann U., Perera L., Berkowitz M.L., Darden T., Lee H., Pedersen L.G. (1995). A smooth particle mesh Ewald method. J. Chem. Phys..

[29] Hubbard S.J., Thornton J.M. (1993). Naccess.

[30] Koromyslova A.D., Chugunov A.O., Efremov R.G. (2014). Deciphering fine molecular details of proteins’ Structure and function with a protein surface topography (PST) Method. J. Chem. Inf. Model..

