# Supplementary material for: Structural Analysis and Activity Correlation of Amphiphilic Cyclic Antimicrobial Peptides Derived from the [W4R4] Scaffold
Source: Molecules. 2023 Dec 12;28(24):8049. doi: 10.3390/molecules28248049 (PMC10745345; doi:10.3390/molecules28248049)
Supplement: Supplementary file 1 [file molecules-28-08049-s001.zip › molecules-2755671-supplementary.pdf]

Supplementary Materials

Structural Analysis and Activity Correlation of Amphiphilic Cyclic Antimicrobial Peptides Derived from the [W_4R_4] Scaffold

Shaima A. El-Mowafi [1,2,†], Anastasia G. Konshina [3,†], Eman H. M. Mohammed [1,4], Nikolay A. Krylov [3], Roman G. Efremov [3,5,*] and Keykavous Parang [1,*]

[1] Center for Targeted Drug Delivery, Department of Biomedical and Pharmaceutical Sciences, Chapman University School of Pharmacy, Harry and Diane Rinker Health Science Campus, Irvine, CA 92618, USA; sa.el-mowafi@nrc.sci.eg (S.A.E.-M.); emohammed@chapman.edu (E.H.M.M.)
[2] Peptide Chemistry Department, National Research Centre, Dokki, Cairo 12622, Egypt
[3] M.M. Shemyakin & Yu.A. Ovchinnikov Institute of Bioorganic Chemistry, Russian Academy of Sciences, Miklukho-Maklaya Street, 16/10, 117997 Moscow, Russia; konnij@gmail.com (A.G.K.); krylovna@gmail.com (N.A.K.)
[4] Department of Chemistry, Faculty of Science, Menoufia University, Shebin El-Koam 51132, Egypt
[5] Department of Applied Mathematics, National Research University Higher School of Economics, Myasnitskaya ul. 20, 101000 Moscow, Russia
* Correspondence: r-efremov@yandex.ru (R.G.E.); parang@chapman.edu (K.P.); Tel.: +7-903-743-16-56 (R.G.E.); +1-714-516-5489 (K.P.)
† These authors contributed equally to this work.

Table S1. MD simulations of the cyclic peptides in the presence of DOPC/DOPG bilayer.

peptides	200 ns[1] Nrun[2]	200 ns[1] A-mode[3]	400 ns Nrun	400 ns A-mode	μs[5]-long Nrun	μs[5]-long A-mode	Nrun in which A-mode occurs during the first 200ns/ 200-400ns/ >400ns
**[W_4R_4(DKP)]**	8	0	2	w1(1)<br>*locked:*[4]<br>w2(9)	2	w1(2)<br>raw(3)	2/1/-
**[W_4R_4]**	9	0	1	w1(3)	2	w2(1)<br>raw(2)	3/1/-
**[W_4R_5]**	10	w2(2)<br>w2(4)	4	raw(1)<br>w1(3)<br>raw(7)<br>raw(8)	2	w1(6)<br>raw(5)	6/2/-
**[W_5R_4]**	6	0	4	w1(1)<br>*no insertion*:<br>(3,5,6)	2	raw(2)<br>raw(4)	2/-/1

[1]Length of MD trajectory; [2]Number of MD runs; [3]MD runs, in which the peptide inserts to the membrane by its hydrophobic Trp-motif (A-mode: residues 5-8/5-9 for 9-mer peptides) are indicated: starting structure and MD trajectory number corresponding to that shown in **Fig. 4A** (in parentheses). For every peptide, three starting structures were used: ″raw″- model is constructed in Maestro program and close to the structure of an "ideal ring" ; "w1″and ″w2″ – representatives of the two most populated clusters of peptide conformations (from MD simulations of the peptide in water); [4]*Locked*″- the membrane-bound state characterized by the partial insertion of Trp-motif and location of DKP-moiety at the water-bilayer interface;[5]1 and 2 μs for [W_4R_4] / [W_4R_4(DKP)] and [W_4R_5] / [W_5R_4] peptides, respectively.

Table S2. Structuring of the peptides in water and water-membrane environments as probed by MD simulations: occurrence (%MD) of bends close to β-turn[1].

**[W$_5$R$_4$]**		**[W$_4$R$_5$]**		*$C_\alpha(i)$ - $C_\alpha(i+3)$*	**[W$_4$R$_4$]**		**[W$_4$R$_4$(DKP)]**	
0,05	0,00	0,04	0,31	**R1-W4**	0,00	0,07	0,04	0,00
0,30	0,15	0,26	0,25	**R2-W5**	0,38	0,16	0,30	0,00
0,00	0,31	0,41	0,46	**R3-W6**	0,02	0,05	0,41	0,24
0,99	0,66	0,18	0,00	**R4-W7**	0,58	0,68	0,44	0,32
0,36	0,02	0,26	0,50	**W5-W8**	0,02	0,24	0,47	0,69
0,10	0,00	0,28	0,14					
0,12	0,04	0,18	0,00	**W6-R1**	0,02	0,00		
0,71	0,56	0,51	0,57	**W7-R2**	0,99	0,75		
0,26	0,32	0,32	0,28	**W8-R3**	0,03	0,23		
water	*water-membrane*	*water*	*water-membrane*		*water*	*water-membrane*	*water*	*water-membrane*

[1]*Occupancies of the corresponding conformational states were estimated by calculating the distances between C_α- atoms of the residues: i, i+3 and the applied cutoff <7 Å.*

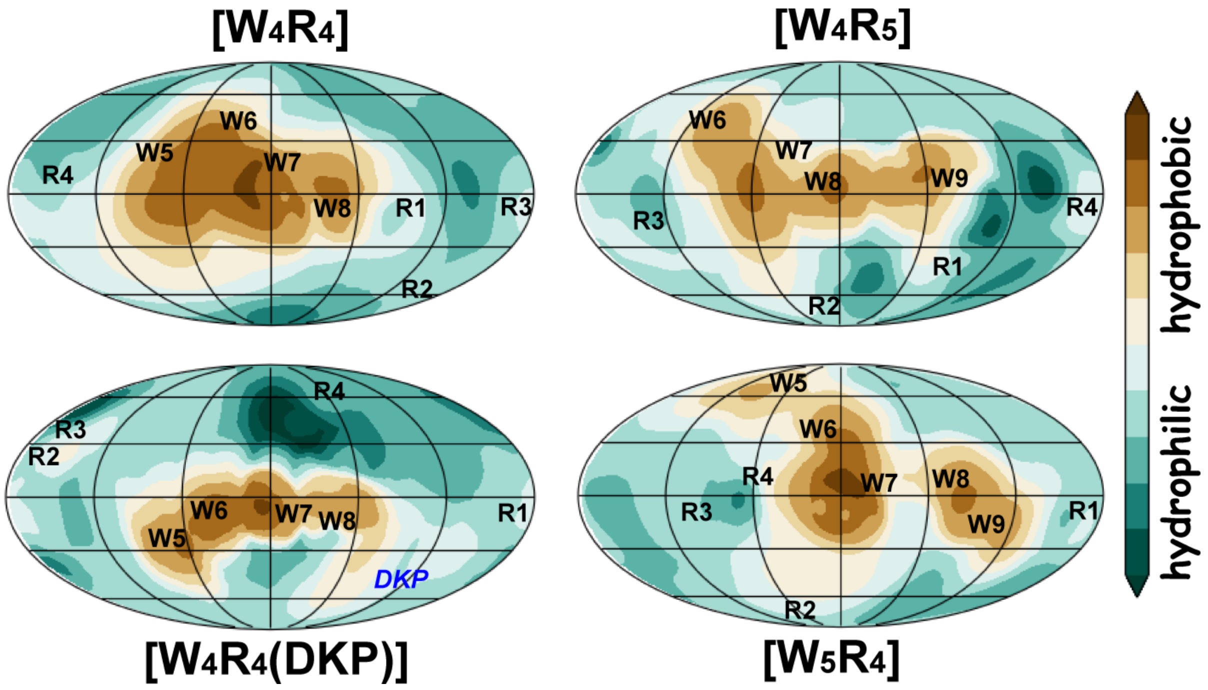

Figure S1. Distribution of hydrophobic / hydrophilic properties on the molecular surface of the peptides [W_4R_4], [W_5R_4], [W_4R_5], and [W_4R_4(DKP)]. Molecular hydrophobicity potential (MHP) values on the protein surface are color-coded according to the scale on the right. MD-averaged MHP spherical projection maps depict entire surfaces of the peptides. The maps were generated using the Protein Surface Topography technique (1). All peptides have a distinct hydrophobic pattern on their surface, consisting of tryptophan residues. The DKP moiety of [W_4R_4(DKP)] forms a weakly polar/apolar zone next to the apolar Trp-motif. Projections of centers of mass of residues (including DKP ring) are labeled. MD-conformations of the peptides were taken from the last 500 ns of equilibrated µs-long MD simulations in a water-membrane environment.

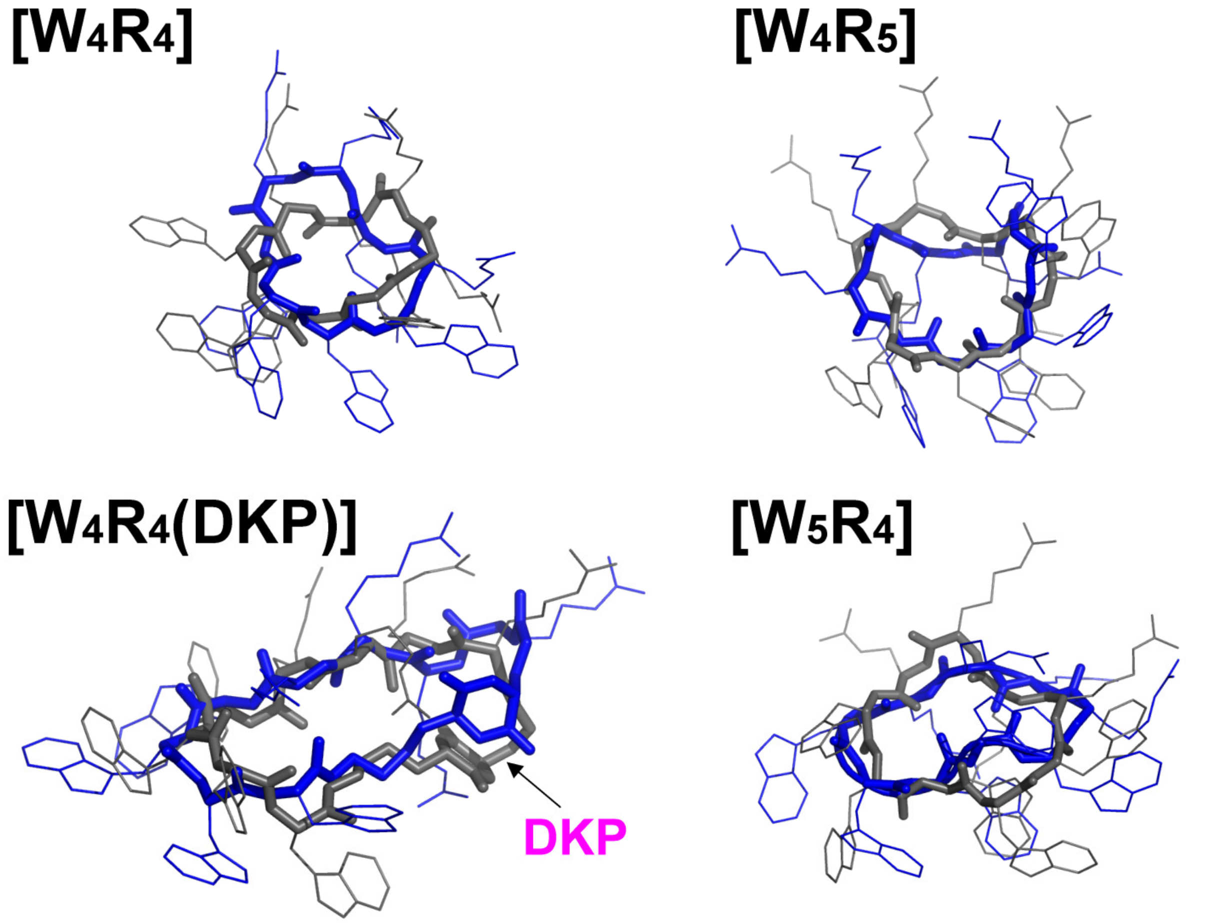

Figure S2. μs-Long MD simulations: different conformations of membrane-embedded states of the peptides [W_4R_4], [W_5R_4], [W_4R_5], and [W_4R_4(DKP)]. For each peptide, superposed spatial structures of the membrane-bound states are taken from the final equilibrated parts of two independent MD simulations. Thick backbone and thin side chain atoms of a peptide are shown in blue and grey for the starting water models and for the starting "ring" conformations, respectively.

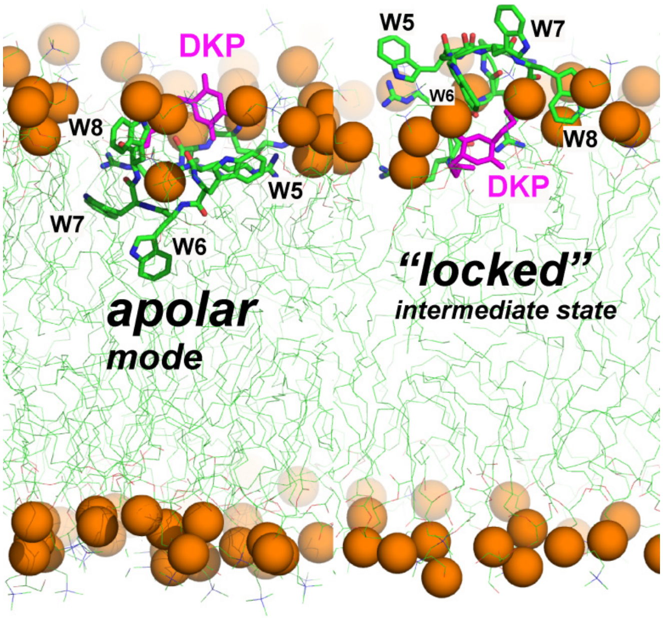

Figure S3. Two membrane binding modes (*apolar* and *locked*) of the peptide [W_4R_4(DKP)] that are potentially important for its membrane activity. Phosphorus atoms of lipids are indicated with golden spheres, while the peptide and lipid molecules are shown in stick and thin lines, respectively. DKP moiety is colored in magenta. Water molecules are not shown for clarity.

REFERENCES

1. Koromyslova, A.D.; Chugunov, A.O.; Efremov, R.G. Deciphering Fine Molecular Details of Proteins' Structure and Function with a Protein Surface Topography (PST) Method. *J. Chem. Inf. Mod.* **2014**, *54*, 1189-1199.
